# mRNA Transfection-Induced Activation of Primary Human Monocytes and Macrophages: Dependence on Carrier System and Nucleotide Modification

**DOI:** 10.1038/s41598-020-60506-4

**Published:** 2020-03-06

**Authors:** Hanieh Moradian, Toralf Roch, Andreas Lendlein, Manfred Gossen

**Affiliations:** 1 Institute of Biomaterial Science, Helmholtz-Zentrum Geesthacht, 14513 Teltow, Germany; 2 Berlin-Brandenburg Center for Regenerative Therapies (BCRT), 13353 Berlin, Germany; 3 Institute of Biochemistry and Biology, University of Potsdam, 14476 Potsdam, Germany; 4 Charité – Universitätsmedizin Berlin, corporate member of Freie Universität Berlin, Humboldt-Universität zu Berlin, and Berlin Institute of Health, Berlin-Brandenburg Center for Regenerative Therapies, Berlin, Germany; 5 Center for Translational Medicine, Medical Department I, Marien Hospital Herne, University Hospital of the Ruhr-University Bochum, Herne, Germany

**Keywords:** RNA, Gene delivery

## Abstract

Monocytes and macrophages are key players in maintaining immune homeostasis. Identifying strategies to manipulate their functions via gene delivery is thus of great interest for immunological research and biomedical applications. We set out to establish conditions for mRNA transfection in hard-to-transfect primary human monocytes and monocyte-derived macrophages due to the great potential of gene expression from *in vitro* transcribed mRNA for modulating cell phenotypes. mRNA doses, nucleotide modifications, and different carriers were systematically explored in order to optimize high mRNA transfer rates while minimizing cell stress and immune activation. We selected three commercially available mRNA transfection reagents including liposome and polymer-based formulations, covering different application spectra. Our results demonstrate that liposomal reagents can particularly combine high gene transfer rates with only moderate immune cell activation. For the latter, use of specific nucleotide modifications proved essential. In addition to improving efficacy of gene transfer, our findings address discrete aspects of innate immune activation using cytokine and surface marker expression, as well as cell viability as key readouts to judge overall transfection efficiency. The impact of this study goes beyond optimizing transfection conditions for immune cells, by providing a framework for assessing new gene carrier systems for monocyte and macrophage, tailored to specific applications.

## Introduction

Innate immune cells play an important role in response to pathological conditions and maintaining immune homeostasis^1^. Among them, monocytes and monocyte-derived macrophages have remarkable properties, including their immunomodulatory capacities. Monocytes with various distinct phenotypes are key players of early inflammation. During inflammation, monocytes can dynamically repolarize to different phenotypes in response to local signals, which is thought to be more efficient at resolving tissue homeostasis than the recruitment of other anti-inflammatory and pro-regenerative subsets of monocytes or macrophages^2–4^.

Macrophages themselves have special functions such as phagocytosis of invading pathogens or apoptotic cells, antigen presentation to T cells, elimination of pathogens via releasing reactive oxygen species or proteolytic enzymes, and secretion of pro- or anti-inflammatory signaling molecules to recruit various types of other immune cells^5–9^. Elucidation and manipulation of monocyte and macrophage phenotypes is therefore essential to fully explore their role in immunoregulation. This will benefit not only basic immunological research but also clinical and translational studies^3,10,11^.

For innate immune cell manipulation, transfections have been commonly used to introduce nucleic acids, such as plasmid DNA (pDNA) or small interfering RNA, to induce or inhibit the expression of a target protein, respectively^12–14^. However, initial studies revealed that, particularly for macrophages, transfection is more challenging in comparison to most other primary mammalian cells^15^. The low transfection efficiency in monocytes/macrophages can be attributed to the following reasons. Firstly, there is a very limited chance for pDNA to freely reach the nucleus due to nuclear envelope breakdown during mitosis, since macrophages do not, or hardly, proliferate^16,17^. Secondly, these immune cells are equipped with pattern recognition receptors, which can detect nucleic acids as potential foreign and dangerous viral invaders, and initiate the inflammatory signaling cascade leading to pDNA degeneration or macrophage apoptosis^18^. Hence, finding a robust transfection approach to address these issues is highly demanded.

**Figure 1:**
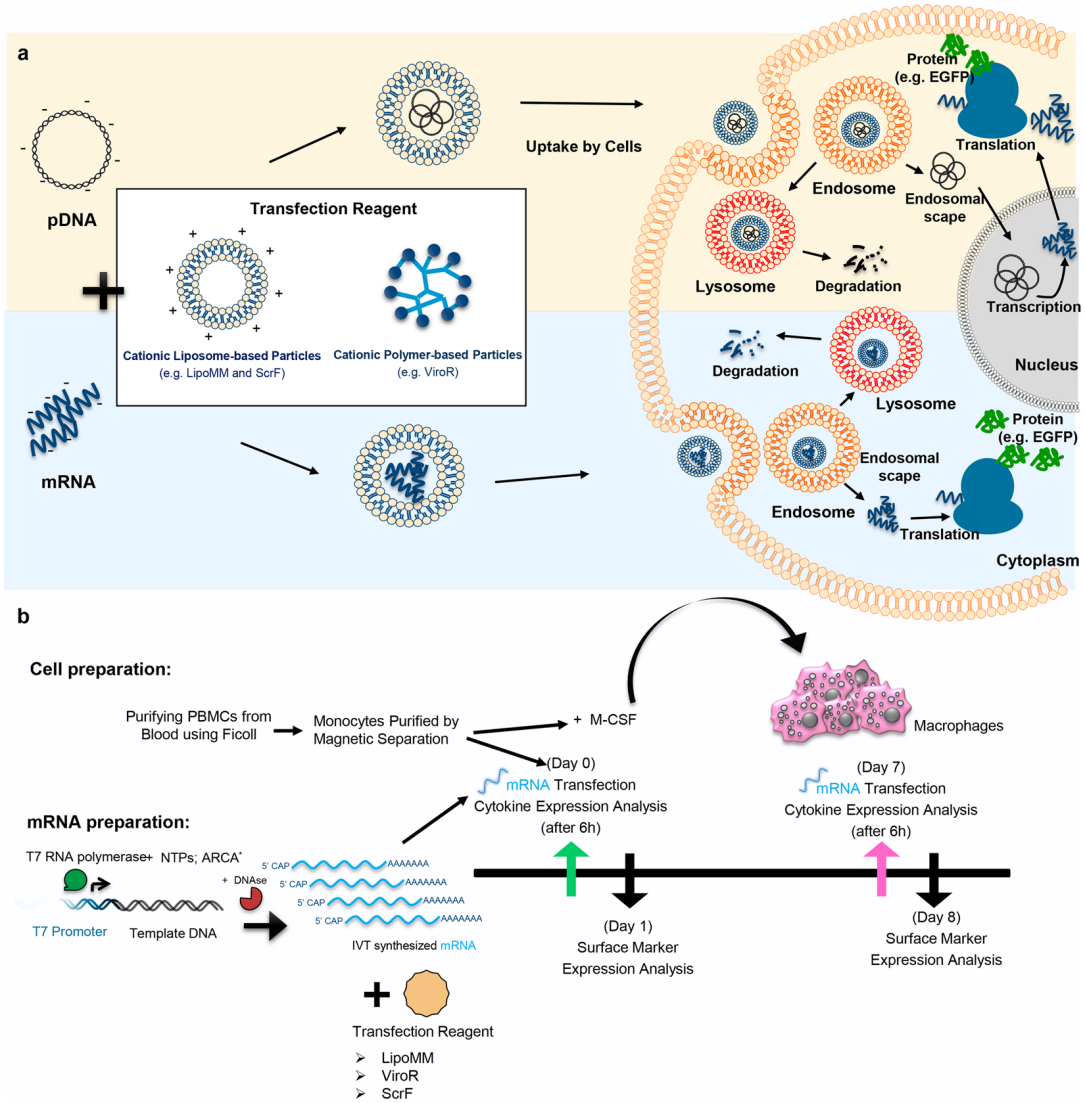
(**a**) Different paths to protein expression upon cells transfection with mRNA or pDNA (**b**) Experimental work flow to isolate and generate primary human monocytes and macrophages and to compare the transfection efficiency of mRNA transfection reagents. LipoMM: Lipofectamine MessengerMax, ScrF: Screen*Fect*, ViroR: Viromer RED, IVT-mRNA: in vitro transcribed mRNA, ARCA: anti–reverse cap analog, EGFP: enhanced green fluorescent protein.

Transfection of messenger RNA (mRNA) is a promising alternative to pDNA or viral vector to achieve target protein expression, particularly in non-proliferative cells such as primary human cells^19,20^. One advantage of mRNA transfection is that there is neither the need for mRNA to enter the cell nucleus, nor the possibility to integrate into the host genomic DNA (Fig. 1a)^21^. Thus, this method can be a proper alternative for transfection of non-proliferative cells^22^, including primary human macrophages and monocytes. Moreover, it will avoid genotoxicity issues associated with chromosomal insertion of DNA vectors in clinical gene transfer applications. In contrast to most pDNA transfections and viral transduction protocols, mRNA transfection will result in a transient, non-stable gene expression. However, transient expression is beneficial for several “hit-and-run” applications, including current differentiation protocols^23,24^.

The mRNA gene delivery technology made significant progress after overcoming commonly known issues related to mRNA, such as susceptibility to degradation by RNases in surrounding media before reaching the target cell^25^. Many parameters of *in vitro* transcription technology have been evaluated and optimized to prevent mRNA degradation, improve translation efficiency, and reduce unspecific immunogenicity upon transfection^19^.

Despite 5′ and 3′-end modifications mimicking natural mRNAs, in vitro transcribed mRNA (IVT-mRNA) can activate immune responses in transfected cells. This effect is often more dramatic for macrophages, which are highly specialized cells for defense against RNA-based viruses and are equipped with numerous receptors including pattern recognition receptors. Toll-like receptors (TLR), particularly endosomal TLR3, 7, and 8, can recognize single- and double-stranded nucleic acids^21^. Therefore, when passing through the endosome, transfected IVT-mRNA could be recognized by immune cells as foreign. However, the immune response can be significantly diminished by utilizing modified nucleotides, as was initially reported by Kariko *et al*. for pseudouridine (Ψ) and 5′-methyl cytidine^26^.

The aim of this study was to set up a robust method for IVT-mRNA transfection in primary human monocytes and monocyte-derived macrophages, while minimizing pleiotropic effects, in particular immune cell activation. We tested three commercially available transfection reagents for mRNA delivery. These included liposomal and polymer-based formulations, as cationic lipid based carriers have different physicochemical properties such as size, shape, and chemical structure compared with polyplexes. These key features not only affect and determine the way they condense and transport their cargo, but also uptake mechanism and subsequent endosomal release, and eventually transfection efficiency. The effects of mRNA modification as well as mRNA concentrations were systematically investigated, using cell viability, transfection efficiency, and the monocyte and macrophage activation as critical readouts. The results of this study provide guidelines for choosing proper mRNA transfection carriers for monocytes and macrophages and highlights the need for not only focusing on gene transfer rates, but also for analyzing cell stress and activation in parallel.

## Results

### Experimental setup

In order to evaluate the effect of different mRNA transfection protocols on monocytes and macrophages, experiments were designed as follows. mRNAs were synthesized using the *in vitro* transcription method, with non-modified or modified nucleotides. Either of three commercially available mRNA transfection reagents, namely the liposomal reagents Lipofectamine MessengerMax (LipoMM) and Screen*Fect* mRNA (ScrF) as well as the polymeric reagent Viromer RED (ViroR) were compared for transfection efficiency; see also Table 1 provided in *Methods*. CD14 positive monocytes purified from peripheral blood mononuclear cells (PBMCs) using magnetic cell sorting, were immediately transfected. A fraction of the CD14 positive monocytes were differentiated into macrophages by cultivation for seven days in the presence of macrophage colony-stimulating factor (M-CSF) and transfected at day 7. Supernatants from monocyte and macrophage cultures were harvested 6 h post transfection and analyzed for the expression of tumor necrosis factor alpha (TNF-α) and interferon beta (IFN-β). One day after transfection, the reporter gene and the CD80 expression was analyzed by flow cytometry. The schematic overview of the experimental workflow is depicted in Fig. 1b.

### Viability of monocytes and macrophages upon mRNA transfection

The forced introduction of nucleic acids in cells often causes substantial stress that might ultimately effect viability. Crucial parameters are type and purity of nucleic acid, the transfection protocol followed and the type of transfection reagent used^27^. The meaningful cell type-dependent optimization of transfection protocols requires that post-transfection phenotypes that are caused by the genetic payload-dependent alteration in the target cells’ transcriptome can be distinguished from side effects of the chosen transfection method or the potential innate immune response of cells upon uptake of exogenous nucleic acids. To this end, we comparatively analysed primary human monocytes and macrophages, treated with different transfection reagents for the introduction of IVT-mRNA encoding enhanced green fluorescent protein (EGFP) to allow for single cell analysis of transfected and non-transfected cells. To address post transfection viability, cells were treated with 4′,6-Diamidino-2-Phenylindole, dihydrochloride (DAPI) immediately prior to the flow cytometric analysis to discriminate live from dead cells. To identify live single cells, a gating strategy as illustrated in Fig. S1 was applied. Cells were first discriminated from debris using forward versus side scatter (FSC vs. SSC) parameters (Fig. S1a). Then, aggregated cells were excluded using FSC-area (FSC-A) against FSC-height (FSC-H) (Fig. S1b) followed by identification of DAPI positive cells, which are considered as dead or apoptotic (Fig. S1c). Among live cells, the amount of EGFP positive cell populations was determined by using untransfected cells as gating control (Fig. S1d).

**Figure 2:**
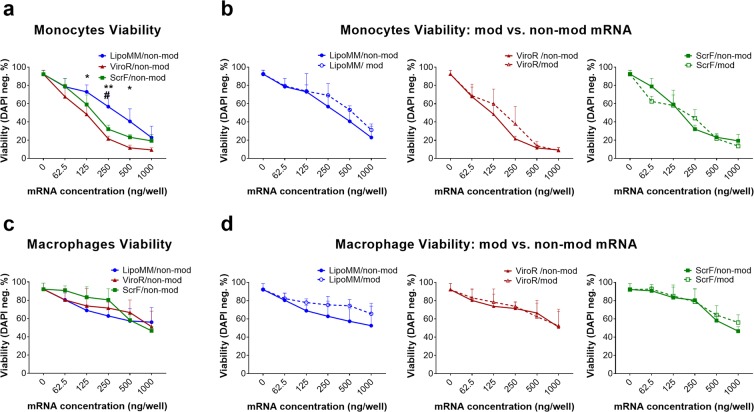
Viability of monocytes and macrophages after mRNA transfection with different mRNA transfection reagents; (**a**) Viability of monocytes transfected with non-modified mRNA using LipoMM (blue), ViroR (red), and ScrF (green); (**b**) monocytes viability after transfection with modified or non-modified mRNA using LipoMM, ViroR, and ScrF. (**c**) Evaluation of macrophages viability after transfection with non-modified mRNA comparing LipoMM, ViroR, and ScrF; viability of macrophages transfected with modified or non-modified mRNA by LipoMM, ViroR, and ScrF. (**d**) Values for no mRNA (0 ng/well) refer to untransfected cells throughout. Values are presented as mean ± standard deviation (SD), n = 3. Error bars indicate SD. Statistical differences in viability is depicted with * for LipoMM vs. ViroR, # for LipoMM vs. ScrF and + for ViroR vs. ScrF. *, ^#^p < 0.05, **p < 0.01.

Monocyte viability was measured after transfection of the three chosen reagents complexed with either modified or non-modified IVT-mRNA. For all transfection reagents, viability decreased with increasing mRNA doses used. LipoMM resulted in significantly higher monocyte viability for most mRNA concentrations, as indicated (Fig. 2a). However, there was no difference between viability of monocytes transfected with ScrF and ViroR (Fig. 2a).

For the macrophage viability, no statistically significant difference between the different transfection reagents was observed when comparing within each dose or average of all doses (Fig. 2c).

The viability of monocytes and macrophages after transfection with mRNA specifically modified with pseudouridine and 5-methyl-cytidine was compared to their viability after transfection with non-modified mRNA for each transfection reagent. In most conditions modified mRNA fails to demonstrate a convincing decrease in cell death when compared to non-modified mRNA for both monocytes and macrophages (Fig. 2b,d), resulting in a lack of statistical significant difference. Surprisingly, monocytes seemed to be more vulnerable to increasing mRNA doses than macrophages, when comparing the overall decrease of viability between monocytes and macrophages (shown in Fig. 2a,c, respectively). For instance, the significant viability decrease was observed in monocytes at 250 ng/well for LipoMM, 62.5 ng/well for ViroR and 125 ng/well for ScrF; whereas, a significant drop in viability of macrophages was only seen above 500 ng/well for all transfection reagents.

### Transfection efficiency in primary human monocytes and macrophages

**Figure 3:**
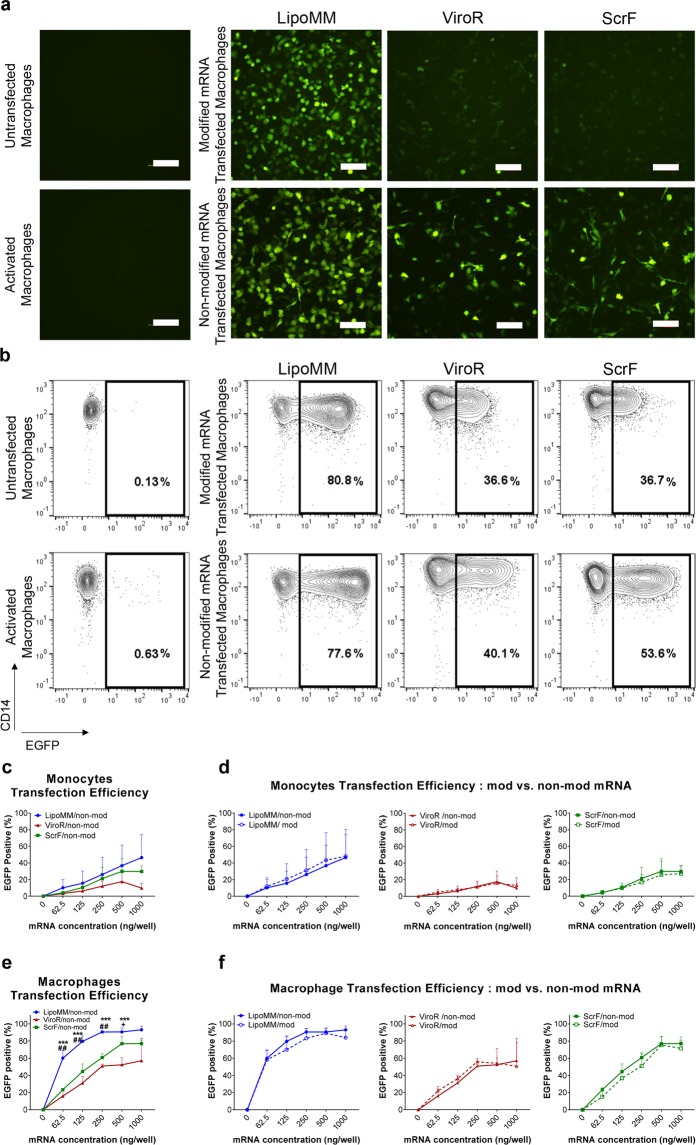
Transfection efficiency for monocytes and macrophages comparing LipoMM, ViroR, and ScrF; (**a**) Representative fluorescent microscopy images (bar = 100 µm) and (**b**) contour plots indicating EGFP expression of macrophages transfected with 125 ng/well of modified and non-modified mRNA via LipoMM, ViroR, and ScrF. (**c**) Quantification of EGFP positive cells as indicator of transfection efficiency for non-modified mRNA transfected monocytes and comparison of monocytes transfected with modified or non-modified mRNA using LipoMM, ViroR, and ScrF (**d**). The same comparison was performed for macrophages transfected with non-modified mRNA (**e**) and for macrophages transfected with modified or non-modified mRNA using LipoMM, ViroR, and ScrF (**f**). Values for no mRNA (0 ng/well) refer to untransfected cells throughout. “Activated” refers to cells treated with lipopolysaccharide (LPS) (2 µg·mL^−1^) and interferon gamma (IFN-γ) (10 ng·mL^−1^) for 24 h. Statistical differences in transfection efficiency is depicted with * for LipoMM vs. ViroR, # for LipoMM vs. ScrF and + for ViroR vs. ScrF. ^+^p < 0.05, ##p < 0.01, ***p < 0.001; Values are presented as mean ± SD, n = 3. Error bars indicate SD.

EGFP was used as a reporter protein to monitor mRNAs transfection efficiency in monocytes and macrophages, which was initially visualized by fluorescent microscopy (Fig. 3a) and subsequently quantified by flow cytometry (Fig. 3b–f). A substantial number of EGFP expressing macrophages could be microscopically observed already for 125 ng mRNA per well, especially when the transfection was performed with LipoMM (Fig. 3a). The numbers of EGFP expressing macrophages after transfection with ViroR and ScrF were clearly lower in comparison to LipoMM. The use of modified mRNA seems to decrease the number of EGFP expressing macrophages, at least for ViroR and ScrF (Fig. 3a). Besides, morphology of transfected macrophages was evaluated using phase contrast microscopy, which indicated morphologically heterogeneous population of cells within various groups compared to untransfected and activated cells (Fig. S2). Moreover, non-modified mRNA resulted in higher EGFP intensity, when compared to modified mRNA for all three transfection reagents (Fig. 3b).

Quantification by flow cytometry over all mRNA doses confirmed a higher frequency of EGFP positive cells among macrophages transfected with mRNA complex with LipoMM comparing to ViroR and ScrF (Fig. 3b,e). No statistical differences were observed when the transfection efficiency was compared for modified and non-modified mRNA (Fig. 3f).

To investigate the dose-dependence of the transfection efficiency for monocytes, the frequency of EGFP positive cells was determined after transfection with increasing amounts of mRNA. The flow cytometry quantification of EGFP expressing monocytes and macrophages revealed increasing transfection efficiency as measured by percentage of EGFP positive cells when increasing amounts of mRNA were applied to the cells. Non-modified mRNA transfection via LipoMM resulted in consistently highest efficiency for both types of immune cells, even more than 80% for macrophages, followed by ScrF and ViroR (Fig. 3c,e). As for the microscopic analysis, only minor differences between the use of modified versus non-modified mRNA was obvious for both monocytes and macrophages (Fig. 3d,f, respectively), at least when quantifying the percentage of EGFP positive cells (see above). Noteworthy, the mRNA dose/response mostly points at saturation effects, i.e. expression plateaued out at high mRNA doses. Overall, it is apparent that macrophages are more efficiently to transfect with mRNA when compared to monocytes.

### Monocytes and macrophages activation

**Figure 4:**
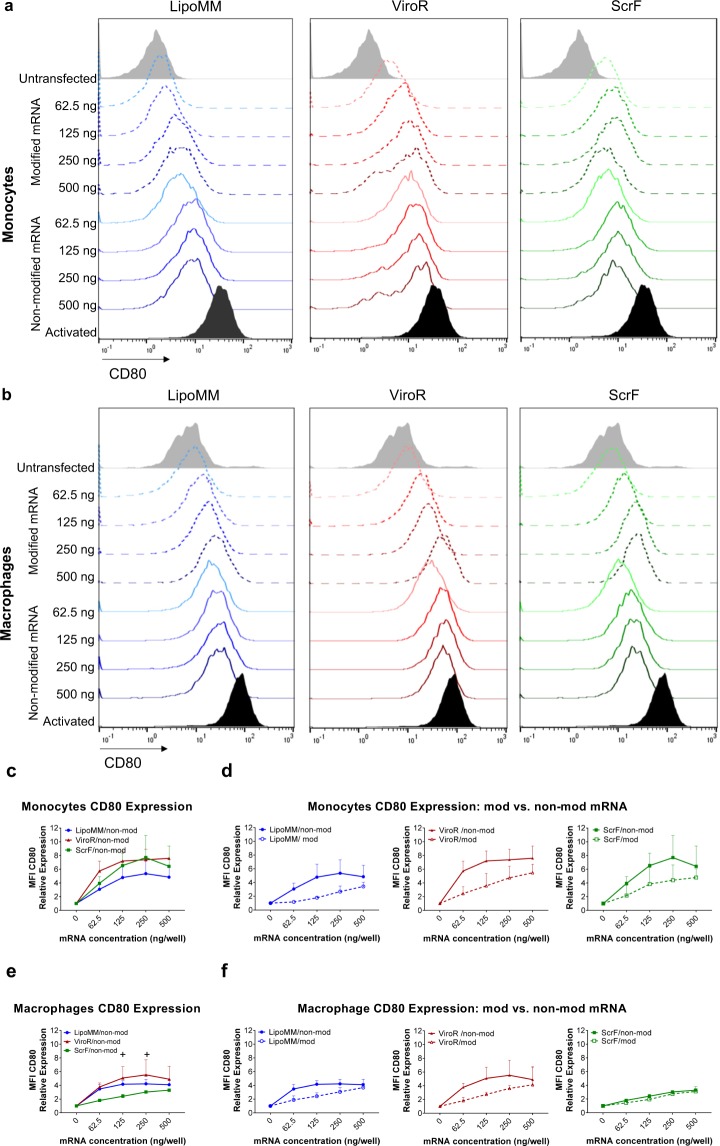
Activation of monocytes and macrophages assessed via CD80 expression; Histograms of CD80 expression in (**a**) monocytes and (**b**) macrophages transfected with modified (dashed lines) and non-modified mRNA (solid line) using LipoMM, ViroR, and ScrF. (**c**) Mean fluorescent intensity (MFI) of CD80 normalized to untransfected cells in monocytes transfected with non-modified mRNA using three transfection reagents. CD80 expression in monocytes transfected with either modified or non-modified mRNA using LipoMM, ViroR, and ScrF (**d**). The same assessment performed for macrophages to compare activation caused by three transfection methods (**e**), or modified versus non-modified mRNA transfected by LipoMM, ViroR and ScrF (**f**). Values for no mRNA (0 ng/well) refer to untransfected cells throughout. “Activated” refers to cells treated with LPS (2 µg·mL^−1^) and IFN-γ (10 ng·mL^−1^) for 24 h. Statistical differences in activation levels are depicted with + for ViroR vs. ScrF. ^+^p < 0.05, **p < 0.01, ***p < 0.001; Values are presented as mean ± SD, n = 3. Error bars indicate SD.

Activation of immune cells such as monocyte or macrophages is a natural process, in which cells acquire pro-inflammatory functions associated with the expression of characteristic cell surface molecules such as CD80 and releasing inflammatory mediators such as TNF-α. Monocytes and macrophages can be activated by lipopolysaccharides (LPS, also known as endotoxin), which can be further potentiated by interferon gamma (IFNγ)^28^. Cell stimulation also occurs in artificial *in vitro* culture settings, such as introduction of external synthetic nucleic acids. Here we investigated whether mRNA transfection can trigger monocyte and macrophage activation. Part of the cell activation by exogenous nucleic acids is based on antiviral response mechanisms of innate immune cells, resulting in the expression of antiviral response molecules, such as cytokine IFN-β. Accordingly, LPS + INF-γ-triggered activation was selected as positive control due to its capacity to induce CD80 and TNF-α expression. Cells treated with the three different transfection reagents were analysed for CD80 expression by flow cytometry. The level of CD80 expression of cells transfected with increasing amounts of modified and non-modified mRNA doses using the three different transfection reagents, LipoMM, ViroR, and ScrF, is illustrated as histograms for monocytes and macrophages (Fig. 4a,b). In general, higher amounts of mRNA resulted in increasing activation levels for all three transfection reagents. However, modified mRNAs triggered less CD80 expression in monocytes and macrophages compared to non-modified mRNA in all conditions (Fig. 4a,b).

Monocytes transfected with LipoMM induced the lowest activation compared to ViroR and ScrF (Fig. 4c). Whereas, the minimum CD80 expression was observed in macrophages transfected with ScrF (Fig. 4e). The polyplex transfection reagent, ViroR, turned out to elicit the highest monocyte and macrophage activation for all mRNA doses (Fig. 4c,e). For monocytes and macrophages, the results also indicated that mRNA modification resulted in substantially less cell CD80 expression when compared to non-modified mRNA (Fig. 4d,f). One exception was the low-level activation of macrophages transfected with ScrF (see above), which was similar for modified and non-modified mRNA (Fig. 4f). Comparing the slope of the CD80 dose/response in monocyte versus macrophages revealed that monocytes activation was more responsive to increasing mRNA doses than in macrophages (Fig. 4c,e).

**Figure 5:**
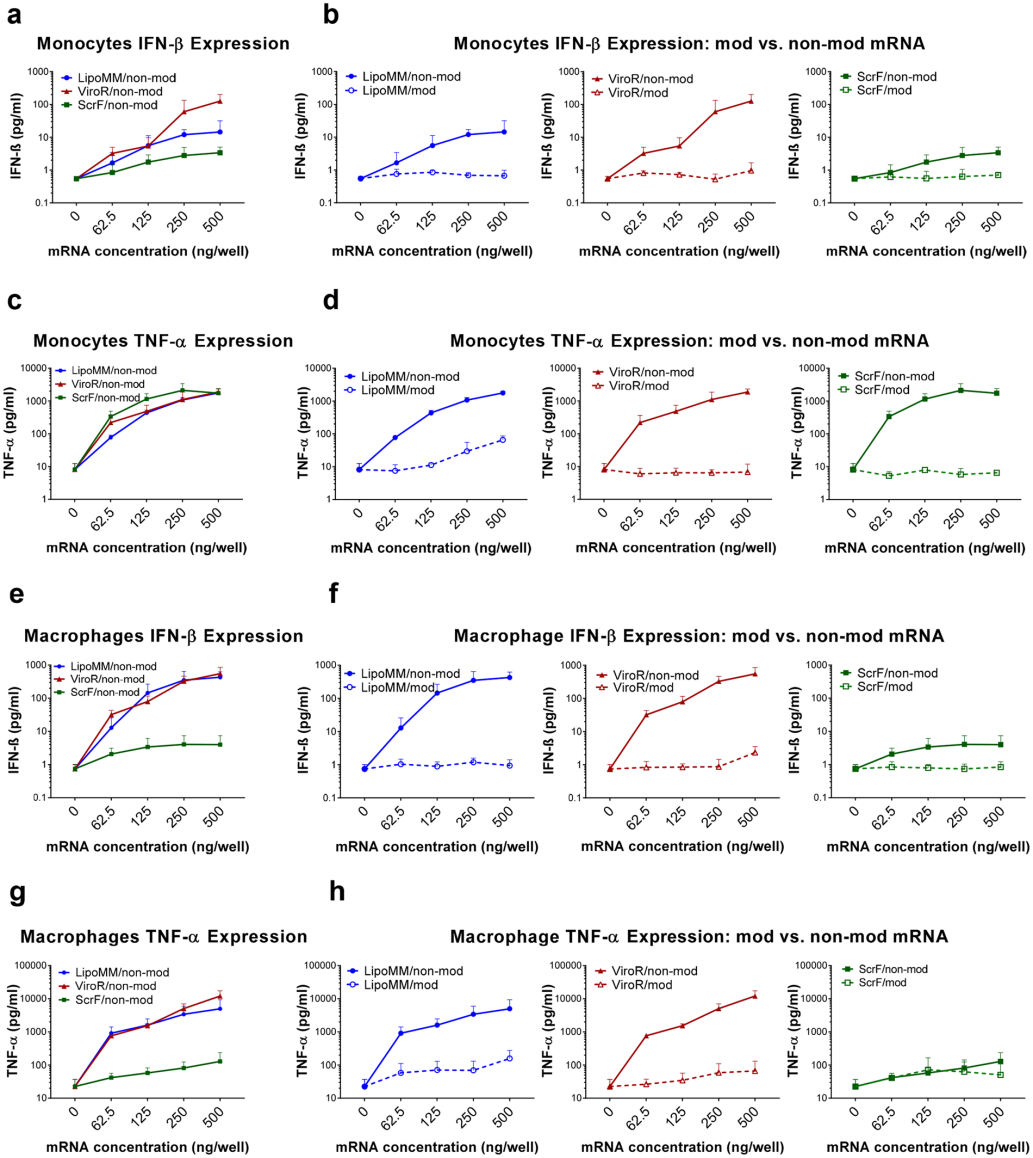
Evaluation of cytokine secretion in transfected monocytes and macrophages; Quantification of IFN-β secreted by monocytes transfected with non-modified mRNA using the three different transfection reagents (**a**) and comparison of IFN-β secretion in monocytes between modified (dashed lines) and non-modified mRNA (solid line) transfected by LipoMM, ViroR, and ScrF (**b**). Quantification of TNF-α secreted by monocytes transfected with non-modified mRNA using three transfection reagents (**c**) and comparison of TNF-α secretion in monocytes between modified (dashed lines) and non-modified mRNA (solid line) (**d**). The analogous assessment as shown in (**a**,**b**) for monocytes performed for macrophage IFN-β secretion secreted in response to the three transfection methods (**e**,**f**). The analogous assessment as shown in (**c**,**d**) for monocytes performed for macrophage TNF-α secretion induced via three transfection reagents (**g**,**h**). All cytokines were measured 6 h after transfection; Values for no mRNA (0 ng/well) refer to untransfected cells throughout. Values are presented as mean ± SD, n = 3. Error bars indicate SD.

When analyzed under the same transfection conditions for the secretion of TNF-α and IFN-β, both cell types overall responded to increasing concentrations of lipo-/polyplex in a dose-dependent manner (Fig. 5). However, while this activation was very modest for modified IVT-mRNA, the use of non-modified IVT-mRNA led to a dramatic increase in cytokine levels, up to two orders of magnitude.

**Figure 6:**
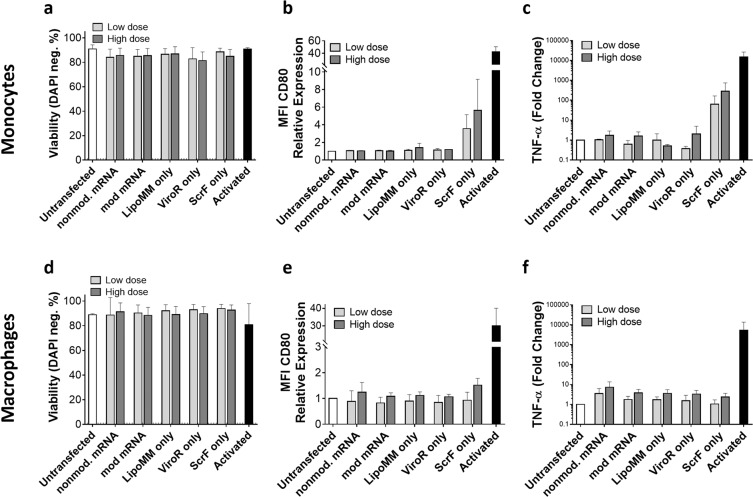
The effect of mRNA and carrier reagent only on viability (**a**), CD80 expression (**b**), and TNF-α secretion (**c**) in monocytes transfected with low dose equivalent to 62.5 ng/well and high dose equivalent to 250 ng/well mRNA condition. In the same way, viability (**d**), CD80 expression (**e**), and TNF-α secretion (**f**) measured in macrophages. Cells were evaluated 24 h after transfection. Values are presented as mean ± SD, n = 3. Error bars indicate SD.

Lastly, we addressed the question if the contact with, or uptake of carriers alone (i.e., without being complexed with mRNA) by cells might contribute to their activation. For most of the conditions analyzed, neither monocytes nor macrophages were activated by “transfection reagent-only”, as assessed by CD80 expression and TNF-α secretion, and no effect on viability could be detected. Only for ScrF reagent added to monocytes, CD80 and TNF-α were slightly increased (Fig. 6). We noted a substantial, but transient acidification of the medium after addition of this transfection reagent, and monocytes might be particular sensitive to these conditions. Overall, increasing mRNA doses always increased cell activation both for monocytes and macrophages, particularly for non-modified mRNA.

### Activation of macrophages transfected with mRNA coding mCherry vs. EGFP

**Figure 7:**
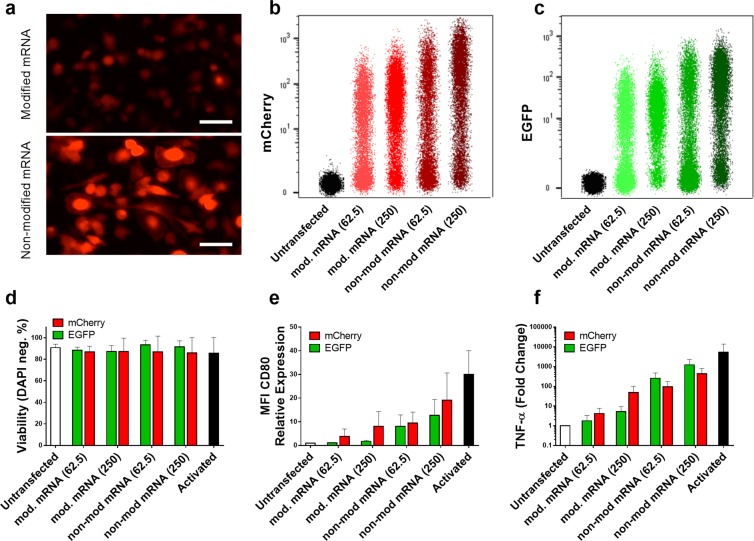
Macrophages transfected with modified or non-modified mRNA encoding for either EGFP or mCherry. mCherry expression was evaluated via fluorescent microscopy (**a**), and flow cytometry (**b**) which was measured in parallel with similar doses (62.5 and 250 ng/well) for EGFP (**c**). Viability of transfected cells was measured side by side (**d**), which shows no significant reduction compared with untransfected cells. Immune activation was assessed via CD80 expression normalized to untransfected cells (**e**) and TNF-α secretion also normalized to untransfected cells (**f**). No difference in CD80 expression was observed between EGFP and mCherry neither for modified nor for non-modified mRNA at lower dose (62.5 ng/well). All parameters have been measured 24 h after transfection. Values are presented as mean ± SD, n=3. Error bars indicate SD.

Certain levels of cell stimulation persisted, despite reduced cell activation for modified mRNA, even for cells transfected with the lowest amount of mRNA for all three transfection reagents. Some studies attribute immune stimulation to the expression of EGFP protein^29–32^. To elucidate if EGFP protein contributes to cell activation, another fluorescent protein with substantially different amino acid sequence was selected. The homology value calculated is only 28.8%. To this end, macrophages were transfected with modified and non-modified mRNA coding for either EGFP or mCherry and the expression of CD80 and TNF-α were evaluated as the marker of cell activation. mCherry expression was validated using fluorescent microscopy (Fig. 7a) and flow cytometry analysis (Fig. 7b,c), which indicated higher mCherry intensity for non-modified mRNA compared with modified mRNA similar to what was observed for EGFP. However, there was no significant decrease in cell viability within different groups (Fig. 7d). The CD80 expression analyzed via flow cytometry illustrated no significant difference in activation of macrophages transfected with mRNA coding EGFP and mCherry within both lower and higher concentrations (Fig. 7e). Consistently, there was no significant difference in TNF-α expression of cells transfected with either of the two evaluated fluorescent proteins (Fig. 7f). Remarkably, the minimum level of CD80 expression and TNF-α secretion was observed for macrophages transfected with lower dose (62.5 ng) of modified mRNA coding EGFP (Fig. 7e,f).

## Discussion

Developing transfection methods for manipulation of macrophage functions would be of utmost interest for basic as well as applied translational researches. In this regard, many studies have investigated different nucleic acid transfection strategies for macrophages, such as gene gun^33^, nucleofection^14,34^, magnetofection^35^, and lipofection^36,37^. However, all of these transfection methods have their limitations due to the resulting poor transfection efficiency, low cell survival, and high rates of immune stimulation of transfected cells^16,17^.

Most of the existing knowledge about monocytes and macrophages comes from either mouse models or cell lines such as murine RAW 264.7 and human THP-1^38^. Neither of these cells represents human monocytes and macrophages properly. However, isolation and culture of primary human monocytes from blood and subsequent *in vitro* differentiation to macrophages is a valuable tool, providing a more precise model for studies focused on macrophages^13^. Thus, to develop a transfection method for primary human cells, which are more relevant for clinical applications, we investigated monocytes isolated from PBMCs and monocyte-derived macrophages.

*In vitro* transcribed mRNA was selected as cargo for this gene transfer study, as it promotes a high level but transient expression of transgenes and lacks genotoxicity. Despite the very high transfection efficiency for higher mRNA doses, up to 90% for macrophages transfected with LipoMM, cell viability under these conditions was low. However, we could achieve over 70% EGFP positive macrophages with no significant impact on their viability. In contrast to pDNA, this high transfection efficiency for the non-proliferative macrophages can at least be partially attributed to the fact that reaching to the cytoplasm is sufficient for mRNA to be expressed. pDNA has to either actively pass the nuclear envelope, or wait for its breakdown during mitosis, a process restricted to proliferating cells. This explanation is consistent with a former study, in which mRNA transfection resulted in over 45% of EGFP expressing cells using two different cell lines with chemically inhibited-proliferation and in non-dividing primary human neurons^22^. In a comparative study, Van De Parre *et al*. also reported a significant difference between mRNA and pDNA transfection efficiency for a murine macrophage cell line^39^. In another study, EGFP coding mRNA nanoparticles, made of Stemfect mRNA transfection reagent, were used for transfection of the JAWSII cell line, primary human and mouse dendritic cells (DCs). This resulted in over 97% transfection efficiency for the cell line, but only around 50% and 60% in human and mouse primary cells^40^. This great gap between the established cell line and the primary cells highlights the importance of using a model, which is more relevant for therapeutic and clinical applications.

Another advantage of using IVT-mRNA is that its *in vitro* production provides the opportunity to incorporate various nucleoside modifications to the transcribed mRNA, and to investigate the impact of these modifications along with non-modified mRNA. Among different modifications, we have chosen the substitution of cytidine and uridine with 5-methyl-cytidine and pseudouridine. Our results showed that the nucleotide modification initiated in general lower activation of monocytes and macrophages upon mRNA transfection in almost all different conditions when compared to non-modified mRNA. The extent of differential immune activation, non-modified vs. modified IVT-mRNA, depended on the chosen readout. Among activation markers, upregulation of CD80 was much less pronounced than that of TNF-α secretion. IFN-β secretion, as an antiviral response marker, was increased by up to two orders of magnitude when using non-modified IVT-mRNA compared to the modified RNA. While cell type and carrier system-dependent differences emerged, they did not consolidate to a clear picture pointing at either lipoplexes or polyplexes as preferred carriers for minimizing immune cell activation. A study by Kariko *et al*., upon evaluation of various modifications, concluded that complete substitution of non-modified nucleosides with 5-methyl-cytidine and pseudouridine, could remarkably reduce DCs cell activation^26^. These DCs were differentiated from primary-human monocytes, the same progenitor cells as for macrophages used in this study. Given the importance of using modified IVT-mRNA to dampen immune cell activation, it is not surprising that efforts continue to find better modifications as well as other approaches to improve the quality of IVT-mRNA^41^. For instance, in a more recent study, Vaidyanathan *et al*. screened various chemical modifications of 5′-CAP and nucleotides as well as transcript sequence optimization for expression of Cas9 protein, as a gene editing tool in CRISPR/Cas9 technology^42^.

At least for the carrier systems and cell types analyzed here, cell activation triggered by intrinsic properties of transfection reagents alone seems to be minor. With one notable exception (ScrF for monocytes), the reagents in absence of mRNA neither decreased viability nor did they upregulate CD80 or TNF-α expression. However, it is noteworthy to point out the limitations of such controls, as “transfection reagent-only” differs in size, and other physical properties such as net charge of the particles, when compared to mRNA-containing lipo- or polyplexes.

The other important factor determining an efficient gene transfection is the type of carrier. Various non-viral gene delivery vehicles made of cationic polymers or lipids with different modifications have been developed^27,43^. However, beyond the transfection of established cell lines, only few of them achieved robust gene delivery in primary cells^22,44^. The number of successful gene transfer systems is even more limited, when delivery of specific cargo such as mRNA is demanded^45^.

Optimized transfection protocols are often a compromise between different requirements, which do not necessarily correlate with each other, including transfection efficiency, viability, and absence of pleiotropic effects^46^. In that sense, LipoMM was distinguished for mRNA transfection of macrophages, in terms of higher transfection efficiency in most transfection conditions in comparison with the two others. This major difference in transfection efficiency could be due to the intrinsic different nature of these particles. Lipoplexes and polyplexes differ in physicochemical characteristics and therefore their function as nucleic acid carrier. Liposomal-based carriers consist of a hydrophilic cationic head group and a hydrophobic tail. The size ratio of these two groups determines the final structure of lipoplex particles upon electrostatic interaction with negatively charged nucleic acid molecules, which can be micellar, vesicle-like bilayer or multilamellar^47^. However, polyplexes can form branched spherical shape, or tubular structure depending of molecular weight, geometry of the cationic polymer, and number of primary amines available at polymer surface^47,48^.

Another important carrier property, which can affect cellular uptake is the surface charge^49^. Lipoplexes are known to have overall positive charge even after complexation with nucleic acids^50^, whereas the polyplex used in this study, ViroR, was reported to have overall neutral charge on the surface upon nucleic acid complexation due to its special chemical structure (information provided by supplier). Complexes with overall positive charge were reported to result in higher transfection efficiency, due to increase in cellular uptake mediated and augmented by initial electrostatic interaction with negatively charged cell membrane proteoglycans^48,51^. However, the disadvantage of having positive surface charge could be the potential aggregate formation in presence of negatively charged serum proteins. Endosomal release is the next important step, which is very crucial for a successful gene transfection. Lipoplexes can escape from endosome by fusion to endosomal membrane and subsequently release their cargo to cytoplasm, due to the presence of hydrophobic tail^47,48^. However, polyplexes cannot harness this mechanism, due to the lack of hydrophobic tail. Instead, the “proton sponge” effect is suggested and widely accepted mechanism to explain their endosomal escape^48^. Another critical step for polyplexes is the release of cargo from the carrier upon successful release to cytosolic space, which can be the limiting factor and hinder successful transfection in case of high molecular weight polycations with high charge density^52^.

Fluorescent proteins have been widely used in gene transfection studies due to their convenient traceability with single cell resolution. Therefore, to ensure that the measured cell activation is primarily attributed to the mRNA-carrier complex and not specific to (over)expression of the chosen reporter protein, another fluorescent marker with different amino acid sequence, namely mCherry, was compared to EGFP. There was no consistent pattern of reporter-specific CD80 or TNF-α stimulation and, most importantly, no differences in viability when comparing EGFP with mCherry. In other words, our results suggest that the observed macrophage activation was not influenced by the type of reporter protein as has been speculated for EGFP, causing cell stimulation by itself in certain experimental settings^31^.

In summary, we systematically investigated responses of primary human monocytes and monocyte-derived macrophages to three widely available mRNA transfection reagents as well as the necessity of mRNA modification. A crucial parameter in our study was the immune activation of cells, which was evaluated and considered as a key factor aside from cells viability and transfection efficiency. Overall, LipoMM turned out to be superior to ViroR and ScrF in terms of higher transfection efficiency and in most cases resulted in higher viability. With regard to immune cell activation we conclude that the use of non-modified IVT-mRNA is the only consistent parameter resulting in low-level activation. By contrast, no clear picture emerged in this study whether the use of lipoplexes or polyplexes would be of principle advantage. Preferences would have to be established depending on the specific cell type and actual reagent considered. Despite the success of lipoplex-based transfection reagents, the future perspective on exploiting mRNA technology in the biomedical and translational researches, like the emergence of transcript-activated matrixes (TAMs)^53^, highlights the need for further development of IVT-mRNA polyplex nanoparticles. For instance, ViroR as a commercial polyplex was outperformed here with regard to cell viability and transfection efficiency by the widely used LipoMM, leaving room for further improvement of polyplex carriers, especially given their potential for *in vivo* delivery. Thus, the results presented in this study might serve as a blueprint for the evaluation of any new mRNA carrier system, in particular highlighting the need for a comprehensive evaluation of cellular immune response mechanism.

## Methods

### mRNA synthesis by *in vitro* transcription (IVT)

A plasmid vector, pRNA2-(A)_128_^54^, was used as a template for *in vitro* transcription of mRNA coding EGFP. This plasmid contains a T7 promoter, 5′UTR, coding region for EGFP, tandem of human β-globin 3′-UTRs, and a 128-base polyadenine [poly(A)] sequence facilitating the generation of mRNA encoded with poly(A) tail without a post-transcriptional *in vitro* tailing reaction. The plasmids were first digested downstream of the poly(A) site using BspMI enzyme (New England Biolabs, Ipswich, MA). The digested plasmids were analysed and simultaneously purified by agarose gel electrophoreses and isolation of the IVT template band using a gel extraction kit (MN, Germany). The concentration of the purified fragment was measured using UV/Vis-spectroscopy (NanoDrop 1000 Spectrophotometer; PEQLAB). mRNAs were synthesized using a TranscriptAid T7 High Yield Transcription Kit (K0441, Thermo Scientific) following the manufacturer’s instruction. The 5′ end of mRNA was modified co-transcriptionally with anti–reverse cap analog (ARCA) (Jena Bioscience, Germany)^55^. Chemically modified mRNAs were also generated by complete substitution of uridine and cytidine with 100 mM pseudouridine (Jena Bioscience, Germany) and 5-methyl-cytidine (Jena Bioscience, Germany), respectively. DEPC treated RNase free water and lithium chloride were added to the mRNA products to the final concentration of 2.5 M and the reaction was incubated at −20 °C overnight followed by centrifugation at 13000 g at 4 °C for 30 minutes. Further washing was done using 70 vol% cold ethanol, and final mRNA products were resuspended in UltraPure™ nuclease-free sterile water (Merck Millipore, Germany). All IVT-mRNAs were analysed by denaturing agarose gel electrophoresis for integrity (Fig. S3) and homogeneity and the concentration was determined photospectroscopically.

### Preparation of primary human monocyte and monocyte-derived macrophages

PBMC were isolated from buffy coats (Deutsche Rote Kreuz, Berlin; ethics vote EA2/018/16; Charité University Medicine Berlin) using Ficoll (L6115, Biochrom, Germany) density gradient centrifugation. Monocytes were purified from PBMCs by negative selection using Monocyte Isolation Kit II (Miltenyi Biotec, Germany) according to the manufacturer’s instruction. Monocytes express high levels of CD14^56^. Therefore, the purity of isolated monocytes was evaluated through CD14 expression, measured by flow cytometry (MACSQuant VYB, Miltenyi Biotec) using previously published protocol^57,58^, which in most cases was at least 80%. Upon purification, cells were suspended in pre-warmed very low endotoxin (VLE) RPMI 1640 (FG 1415, Biochrom) supplemented with 10 vol% FBS (Biochrom) and were seeded in 24 well plates (TPP Techno Plastic Product AG, Switzerland) at a density of 5 × 10^5^ cells per well. To avoid unspecific endotoxin mediated cell activation, all solutions used in the monocyte and macrophage assays were evaluated for endotoxin levels using EndoLISA^®^ test (Hyglos, Germany) and only used when the amount of detected endotoxins was below 0.5 EU/mL. Monocytes were either used directly for transfection or further cultivated at 37 °C and 5 vol% CO_2_ for 6–7 days in medium supplemented with 50 ng·mL^−1^ human M-CSF (Miltenyi Biotec) to generate monocyte-derived macrophages. The medium was changed every third day, upon washing vigorously with pre-warmed complete medium to remove non-adherent cells. Cells were cultured in antibiotic-free medium.

### mRNA transfection using three different transfection reagents

The transfection of mRNA was performed using three commercially available transfection reagents including Lipofectamine MessengerMAX (Thermo scientific), Viromer RED (Lipocalyx, Germany), and Screen*Fect* mRNA (InCella, Germany), all of which are, according to the manufacturers, specifically formulated for mRNA transfection. To the extent available through the suppliers, physical and chemical characteristics as well as validated target cells are provided in Table 1.

**Table 1:** Characteristics of the three commercially available mRNA transfection reagents provided by the manufacturer.

Features provided by the manufacturer	LipoMM	ViroR	ScrF
Material’s chemistry	Lipid-based nanoparticle	Polymeric carrier, based on polycationic PEI core, highly substituted with hydrophobic and anionic side chains	Cationic thioether lipids, containing hydrophobic alkyl groups
Specific structural features	Cationic lipids optimized for mRNA delivery application;	Polymers mimicking viral (influenza hemagglutinin) biophysics	Biomimetic lipid-like molecules made by thiol-yne click chemistry
Tested cell types	Primary cell types such as neurons, fibroblast, hepatocytes and Keratinocytes, specifically tested for mRNA CRISPRs	Primary adherent and suspension cells including monocytes and macrophages and stem cells	Many different human and mouse cell lines such as HEK293, NIH3T3, RAW 264.7 and mouse embryonic stem cells (mESC)

LipoMM: Lipofectamine MessengerMax, ScrF: Screen*Fect*, ViroR: Viromer RED.

A premixed concentrated solution containing carrier-mRNA complexes was prepared for each transfection reagent according to the detailed protocol describes as follows. MessengerMAX reagent was diluted in Opti-MEM medium (Gibco® by life technologies^TM^, Germany) at 1:50 volume ratio, and incubated for 10 minutes at room temperature. The equal volume of diluted modified or non-modified IVT-mRNAs in Opti-MEM medium (4 ng·µL^−1^) were subsequently added to MessengerMAX solution. LipoMM-mRNA complex mixtures were incubated for 5 minutes at room temperature and the corresponding volumes to deliver various mRNA doses (62.5, 125, 250, 500, 1000 ng per well) were added to each 24-well.

To prepare ViroR-mRNA polyplexes, mRNA was diluted in 225 µL of provided Viromer RED buffer at 11 ng·µL^−1^. In another tube, a 0.75 µL droplet of Viromer® was placed on the tubes’ wall and immediately mixed with 18 µl of buffer and vortexed for 5 seconds. The mRNA solution was then added to the diluted Viromer RED®, mixed swiftly, and incubated for 15 min at room temperature.

The ScrF-mRNA master solution was prepared as follows. The concentrated Screen*Fect*® mRNA was mixed with the provided dilution buffer (1:20 volume ratio) then combined immediately with the equal volume of mRNA diluted in the same buffer (8 ng·µL^−1^). The resulting solution was mixed by pipetting thoroughly, and incubated for 20 min at room temperature to allow complex formation.

To transfect monocytes immediately after cell purification, upon formation of an mRNA-transfection reagent master mixes, the corresponding amount of mRNA complexes were added to the respective empty well (24-well plate) followed by adding 500 µl of cell suspension. Accordingly, at the end of the differentiation period at day 7, the medium was replaced with warm VLE RPMI supplemented with 10 vol% FBS. After 4 h, the proper amounts of transfection reagent-mRNA complexes were added dropwise to each well of monocyte-derived macrophages. As positive control for immune stimulation, LPS (2 µg·mL^−1^) (Enzo life science, USA) and IFN-γ (10 ng·mL^−1^) (Miltenyi Biotec) were added to the medium of untransfected cells.

### Evaluation of transfection efficiency by fluorescent microscopy

To evaluate the EGFP expression of adherent human macrophages, cells were imaged 24 h after transfection, using a Nikon inverted microscope ELIPSE T*i*-U equipped with long-life mercury light source, Intensilight C-HGFI. The NIS-Elements imaging software package (version 4.51) was used to analyze microscopic images.

### Measurement of cell viability, transfection efficiency, and activation of monocytes and macrophages by flow cytometry

Cells were harvested 24 h after transfection for further staining and analyzed by flow cytometry. Whereas monocytes, could be harvest by pipetting, macrophages had to be dissociated using TrypLE Select (Gibco® by life technologies^TM^, Germany) according to manufacturer’s instruction. To avoid unspecific antibody binding, cells were blocked by incubation with FcR Blocking Reagent (Miltenyi Biotec) for 10 min at 4 °C after washing with flow cytometry washing solution (PBS pH 7.2, BSA, EDTA). Subsequently, cells were stained with antibodies including anti-human CD14-PE-Vio770 (clone TÜK4) (Miltenyi Biotec), and CD80-PE (clone L307.4) (BD Pharmingen™, San Jose, USA) for 10 min at 4 °C using the recommended dilution factor 1:100 (5 µg·mL^−1^ final concentration). After a final washing step with cold flow cytometry washing solution, cells were acquired with MACSQuant VYB® (Miltenyi Biotec). DAPI at a final concentration of 1 µg·mL^−1^, was added to each sample immediately prior to flow cytometric analysis, to discriminate DAPI-negative live cells from DAPI-positive dead cells. All flow cytometric data were analysed using FlowJo software V10.

### Cytokine detection in monocyte and macrophage cell culture supernatants

Monocyte and macrophage culture supernatants were harvested and stored at −20 °C until further usage. The secretion of IFN-β and TNF-α was quantified in thawed supernatants using Bio-Plex^®^ Multiplex Immunoassay System (BioRad, Geramny) according to the manufacturer’s instructions. Briefly, to prepare the standard curves, 50 µL of the reconstituted cytokine standards were added to 150 μL culture medium (the same batch as samples were collected) and eight 4-fold serial dilutions were made. Anti-cytokine coupled beads were diluted in assay buffer and 50 μL were added into each well of the plate. Plates were washed twice, before 50 μL of standard solution or sample supernatants were added. After incubation at 900 rpm for 30 min at room temperature, plates were washed three times with 1x washing buffer. The detection antibodies (20 × stock) were diluted in detection antibody diluent HB, and 25 μL were added into each well followed by an incubation at 900 rpm for 30 min at room temperature. After plates had been washed three times, 50 μL of PE conjugated streptavidin diluted 1:200 were added into each well and incubated at 900 rpm for 10 min at room temperature. After three final washing steps, beads were resuspended in 125 μL assay buffer, shaken at 900 rpm for 30 s and the plates were analyzed using the Bio-Plex^®^ 200 system (BioRad, Germany).

### Comparison of EGFP and mCherry in terms of macrophages activation

The PCR-amplified DNA fragment encoding mCherry was cloned into pRNA2-(A)_128_. Briefly, EGFP coding sequence was replaced with mCherry by digestion of plasmid with *HindIII* and *NotI* restriction enzymes (New England Biolabs), and insertion of mCherry fragment in the vector. mRNA synthesis was performed using the new plasmid, pRNA2-(A)_128_-mCherry, exactly as described for pRNA2-(A)128; see *Methods* section “mRNA synthesis by *in vitro* transcription”. Macrophages were transfected with 62.5 ng and 250 ng mRNA coding EGFP or mCherry by Lipofectamine MessengerMAX (Thermo scientific). Moreover, the homology value of the two proteins amino acid sequence was calculated by NCBI online blast tool.

### Statistics

Data are presented as means± standard deviation (SD) of at least three independent experiments. Normally distributed data of multiple groups were statistically analysed by Two-Way ANOVA, Tukey’s multiple comparison test using GraphPad Prism 7.00 (La Jolla, CA 92037, USA). Statistical significance is considered as p < 0.05.

## Supplementary information

Supplementary Figures.

## Data Availability

The datasets generated during and/or analysed during the current study are available from the corresponding author on reasonable request.
