# Supplementary material for: mRNA Transfection-Induced Activation of Primary Human Monocytes and Macrophages: Dependence on Carrier System and Nucleotide Modification
Source: Sci Rep. 2020 Mar 6;10:4181. doi: 10.1038/s41598-020-60506-4 (PMC7060354; doi:10.1038/s41598-020-60506-4)
Supplement: Supplementary file 1 — Supplementary Figures. [file 41598_2020_60506_MOESM1_ESM.pdf]

mRNA Transfection-Induced Activation of Primary Human Monocytes and Macrophages: Dependence on Carrier System and Nucleotide Modification

Hanieh Moradian[1, 2, 3], Toralf Roch[1,4,5], Andreas Lendlein[1, 2, 3], Manfred Gossen[1, 2, *]

1 Institute of Biomaterial Science, Helmholtz-Zentrum Geesthacht, 14513 Teltow, Germany

2 Berlin-Brandenburg Center for Regenerative Therapies (BCRT), 13353 Berlin, Germany

3 Institute of Biochemistry and Biology, University of Potsdam, 14476 Potsdam, Germany

4 Charité – Universitätsmedizin Berlin, corporate member of Freie Universität Berlin, Humboldt-Universität zu Berlin, and Berlin Institute of Health, Berlin-Brandenburg Center for Regenerative Therapies, Berlin, Germany

5 Center for Translational Medicine, Medical Department I, Marien Hospital Herne, University Hospital of the Ruhr-University Bochum, Herne, Germany.

* Corresponding Author, email address: manfred.gossen@hzg.de

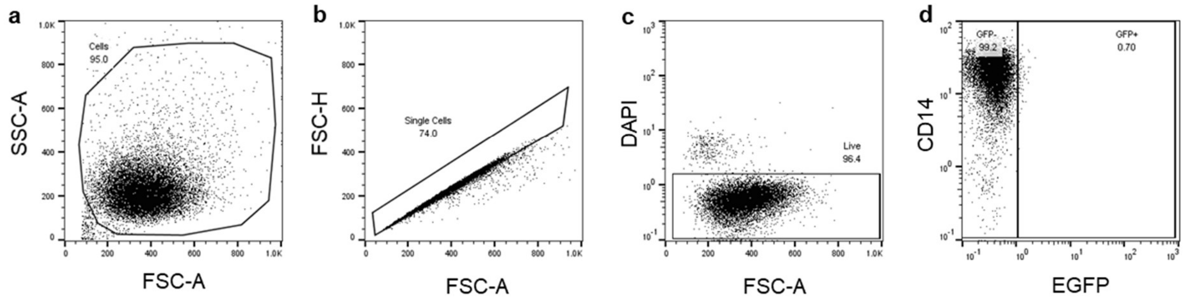

Supplementary Figure 1. Gating strategy for flow cytometry data analysis; cells were discriminated from debris (**a**), followed by exclusion of aggregated cells (**b**). Discrimination of live and dead cells using DAPI as indicator for cell death (**c**), Identification of GFP expressing CD14 positive monocytes and macrophages (**d**)

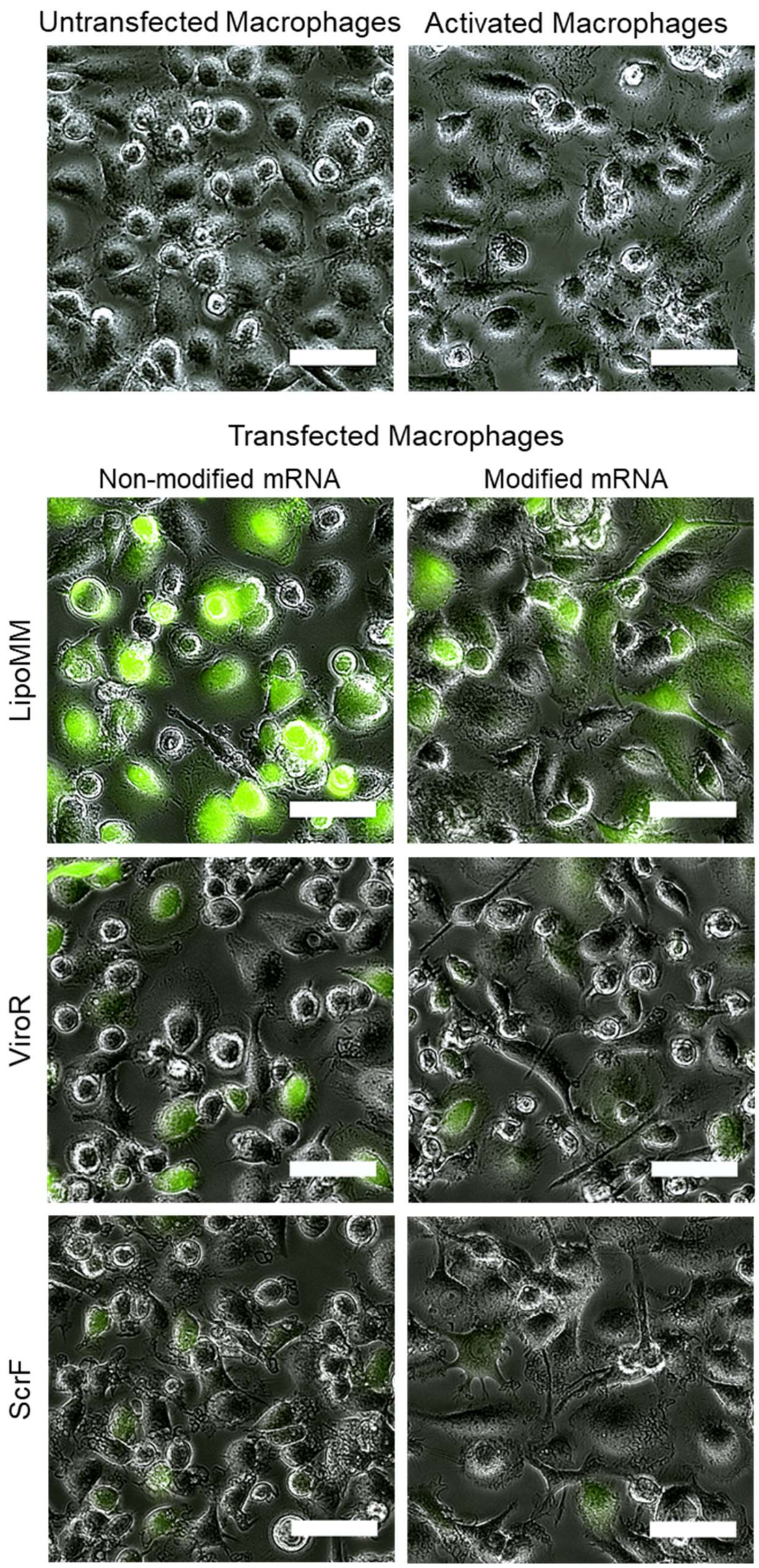

Supplementary Figure 2. Morphological analysis of Macrophages transfected with either modified or non-modified mRNA using three carrier reagents (125 ng/well). Phase contrast merged with EGFP fluorescent images show typical macrophage morphology for transfected as well as untransfected and activated (LPS+IFN-γ) cells (Scale bar=50 µm).

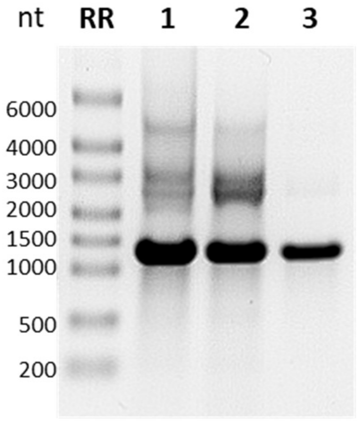

Supplementary Figure 3. Evaluation of IVT-mRNA integrity; mRNA product before (lane 1) and after (lane 2) treatment with DNase, as well as after purification using LiCl (lane 3) shows no residual plasmid template DNA or obvious degradation products. RiboRuler (RR; Thermo Scientific™) was used as size standard. The full-size gel (uncropped) is presented in Supplementary Figure 4.

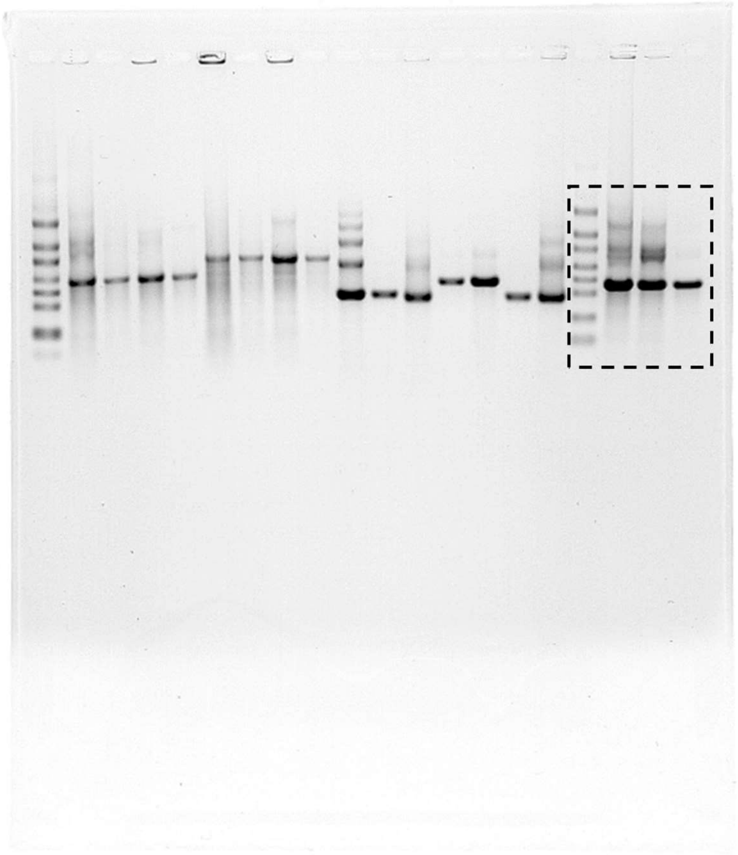

Supplementary figure 4. Area inside the dashed rectangle corresponds to cropped gel picture presented as Supplementary Figure 3. All other lanes are unrelated to this study.
